# Are commercial providers a viable option for clinical bacterial sequencing?

**DOI:** 10.1099/mgen.0.000173

**Published:** 2018-04-05

**Authors:** Kathy Raven, Beth Blane, Carol Churcher, Julian Parkhill, Sharon J. Peacock

**Affiliations:** ^1^​ Department of Medicine, University of Cambridge, Addenbrooke's Hospital, Hills Road, Cambridge CB2 0QQ, UK; ^2^​ Wellcome Sanger Institute, Wellcome Genome Campus, Hinxton, Cambridge, CB10 1SA, UK; ^3^​ London School of Hygiene and Tropical Medicine, London, UK

**Keywords:** whole-genome sequencing, MRSA, clinical application

## Abstract

Bacterial whole-genome sequencing in the clinical setting has the potential to bring major improvements to infection control and clinical practice. Sequencing instruments are not currently available in the majority of routine microbiology laboratories worldwide, but an alternative is to use external sequencing providers. To foster discussion around this we investigated whether send-out services were a viable option. Four providers offering MiSeq sequencing were selected based on cost and evaluated based on the service provided and sequence data quality. DNA was prepared from five methicillin-resistant *Staphylococcus aureus* (MRSA) isolates, four of which were investigated during a previously published outbreak in the UK together with a reference MRSA isolate (ST22 HO 5096 0412). Cost of sequencing per isolate ranged from £155 to £342 and turnaround times from DNA postage to arrival of sequence data ranged from 12 to 63 days. Comparison of commercially generated genomes against the original sequence data demonstrated very high concordance, with no more than one single nucleotide polymorphism (SNP) difference on core genome mapping between the original sequences and the new sequence for all four providers. Multilocus sequence type could not be assigned based on assembly for the two cheapest sequence providers due to fragmented assemblies probably caused by a lower output of sequence data per isolate. Our results indicate that external providers returned highly accurate genome data, but that improvements are required in turnaround time to make this a viable option for use in clinical practice.

## Data Summary

Genomes are deposited in the European Nucleotide Archive under the accession numbers listed in Table S1 (available in the online version of this article) (https://www.ebi.ac.uk/ena).

**Impact Statement**

Whole-genome sequencing is a highly discriminatory method that can define the extent to which bacteria are related. This has major potential for use in infection control and could enhance the accuracy of outbreak detection. One barrier to local implementation is the lack of sequencing instruments in most routine microbiology laboratories. An alternative is to use an external genome sequencing provider. Here, we compared several sequencing providers for price, turnaround time, ease of use and quality of returned data. We found that external providers returned highly accurate genome data, but that improvements are required in turnaround time to make this a viable option for use in clinical practice.

## Introduction

The utility of whole-genome sequencing (WGS) for accurately identifying the population structure and genetic relatedness of pathogenic bacteria has been demonstrated for a range of species [1–4]. Associated with this is growing support for its translation into diagnostic and public health microbiology, including for the investigation of suspected outbreaks. This could improve the accuracy of the current infection control paradigm, in which suspected outbreaks are largely based on epidemiological evaluation of two or more patients positive for the same pathogen to determine whether an opportunity for transmission could have occurred. However, this approach has low sensitivity for transmission detection, probably because patients can be cared for in several wards during the same admission and outbreaks can span extensive time periods and multiple wards and even hospitals [1, 2]. When an investigation suggests that an outbreak is likely, isolates may be typed in-house or by a reference laboratory using a range of methods that generally lack discrimination, may have a long turnaround time and may not be comparable between laboratories [3, 5, 6]. WGS has superior discriminatory power compared to other typing techniques and its utility for the investigation of clinical outbreaks has been confirmed [5, 7]. The generation of the entire genome also has the potential to detect genes encoding antibiotic resistance and virulence factors.

Sequencing instruments are not generally available in the majority of routine microbiology laboratories. The cost (start-up and running), space and expertise required to undertake bacterial WGS to support routine infection control practice are barriers to local implementation. An alternative would be to use one of the increasing number of commercial WGS services available. The aim of the study was to compare a selection of these WGS services for price, turnaround time, ease of use and quality of returned data.

## Methods

### Selecting commercial sequencing providers

In November 2016, we searched the internet for commercial sequencing providers using ‘DNA sequencing’, ‘bacterial’ and ‘service’ as search terms. This resulted in the identification of eight providers, who were contacted and quotes requested for library preparation and paired-end whole genome sequencing of five methicillin-resistant *Staphylococcus aureus* (MRSA) isolates multiplexed in a single run on an Illumina MiSeq instrument. Providers are referred to here as A to H, details of which are shown in Table 1.

**Table 1.** Overview of whole-genome sequence providers

Sequence provider	Country	Cost per sample (£)	Stated turnaround time (days)	Actual turnaround time (days)	Delivery method (as requested)	Method of data return
B	Mainland Europe	155.58	21–35	29	Regular post, room temperature	USB stick in post
C	UK	338.16	7–14	12	Regular post, room temperature	Link to Illumina BaseSpace project
F	UK	336.00	28–42	28	Courier, ice box	Link to ftp site
D	UK	342.00	56–70	63	Courier, ice box	Link to secure company website
E	UK	612.00	21–28	Not applicable	Not applicable	Not applicable
H	Mainland Europe	836.00	28	Not applicable	Not applicable	Not applicable
A	UK	900.00	Not provided	Not applicable	Not applicable	Not applicable
G	Asia	Not available*	Not applicable	Not applicable	Not applicable	Not applicable

*Provider G did not provide a MiSeq service, only HiSeq services.

### Bacterial isolates

The bacterial isolates used for the evaluation were four MRSA isolates sequenced previously as part of an outbreak investigation on a Special Care Baby Unit (SCBU) [8], and the MRSA reference isolate ST22 HO 5096 0412 (accession number HE681097). Three of the four SCBU MRSA isolates [one each from patient (P)14 (genome sequence accession number ERR72246), P15 (accession number ERR108054) and P23 (accession number ERR72247)] were multilocus sequence type ST2371 (a single locus variant of ST22), and were closely related. P14 and P23 had zero single nucleotide polymorphism (SNP) difference in the core genome, and P15 differed from these by five SNPs and had lost an *ermC* plasmid. The fourth SCBU isolate (accession number ERR131801, ST22) was more distantly related and not considered to be part of the outbreak, and is referred to here by the original isolate number SASCBU35. Sequences for these isolates were originally generated using an Illumina MiSeq with 150 bp paired-end reads at the Wellcome Sanger Institute. Their genetic relatedness was assessed in the original study by mapping to the ST22 HO 5096 0412 reference using SMALT (http://www.sanger.ac.uk/science/tools/smalt-0). The SNP calling parameters used were: ≥4 reads matching the SNP, minimum of 2 reads per strand; required read support =0.8; reads must have greater than 90 % match to the reference to be mapped; and no removal of clustered SNPs or genome masking.

### DNA extraction and sequence data analysis

Stored isolates were plated onto Columbia blood agar (Oxoid) and incubated for 24 h in air at 37 °C. A single colony was picked and sub-cultured onto Columbia blood agar and incubated as before. Ten single colonies from each isolate were selected from the purity plate for DNA extraction (ten DNA replicates per isolate). DNA was extracted using the QIAmp mini DNA kit (Qiagen) and the ten replicates for each isolate were then pooled. Aliquots of the pooled samples were sent to each provider, with the exception of provider D which required an EDTA-free elution after sample preparation. DNA quantity was measured using a Qubit 2.0 fluorometer (ThermoFisher Scientific). Adjustments were made to the DNA concentration using DNAse-free water to meet the input DNA requirements of each provider, and samples were sent using the requested method of delivery (Table 1).

Sequence data from each provider were downloaded from either a user-accessed website (providers C, D and F) or a USB-stick (provider B). The original sequence data for the four clinical isolates (P14, P15, P23 and SASCBU35) and the ST22 reference strain (HO 5096 0412) were downloaded from the Sanger database and the European Nucleotide Archive (ENA, http://www.ebi.ac.uk/ena/), respectively. Species identification was performed using Kraken [9]. All sequences were assembled using Velvet [10] and annotated using Prokka [11]. The ST was identified using an in-house script (https://github.com/sanger-pathogens/mlst_check) [12]. Multilocus sequence type (MLST) alleles that could not be identified were investigated further using blast. All sequences were mapped to the ST22 reference strain using SMALT. SNPs were identified using an in-house script (https://github.com/sanger-pathogens/snp-sites). The parameters used to call a SNP were as follows: ≥4 reads matching the SNP, minimum of 2 reads per strand; required read support =0.75; no removal of clustered SNPs or genome masking.

## Results

### Service provision by commercial providers

We initially identified eight commercial providers and obtained quotes for library preparation and whole genome sequencing of five MRSA isolates on a single MiSeq run. Quoted cost ranged from £155.58 to £900 per isolate. Providers with the four lowest quotes were selected for further evaluation. Cost, DNA preparation, postage requirements, turnaround time (from the day of postage/collection by courier) and data format are summarized in Table 1, which shows variation in all of these parameters. The time between posting the sample and receipt by the company was 1–2 days within the UK (both regular post and courier) and 7 days for the service based outside of the UK. The actual median (range) turnaround time was 28.5 days (12–68 days). All companies delivered the data within their pre-defined deadline. Provider B was the cheapest (£155.58 per sample), whilst provider C had the fastest turnaround time (12 days).

### Sequence data metrics

All sequences were correctly assigned as *S. aureus* with greater than 89 % of the reads matching *S. aureus* (Table S1). However, the number of reads classified as other species (indicating possible contamination) varied (Table S1), with provider B having higher levels of putative contamination compared to the others. Provider D indicated that the sequence data for P14 were probably contaminated with *Staphylococcus warneri*, which was consistent with findings from our informatic analysis. We found that 0.11 % of reads from isolate P14-provider D mapped to this species compared to 0 % of reads from other companies.

**Fig. 1.**
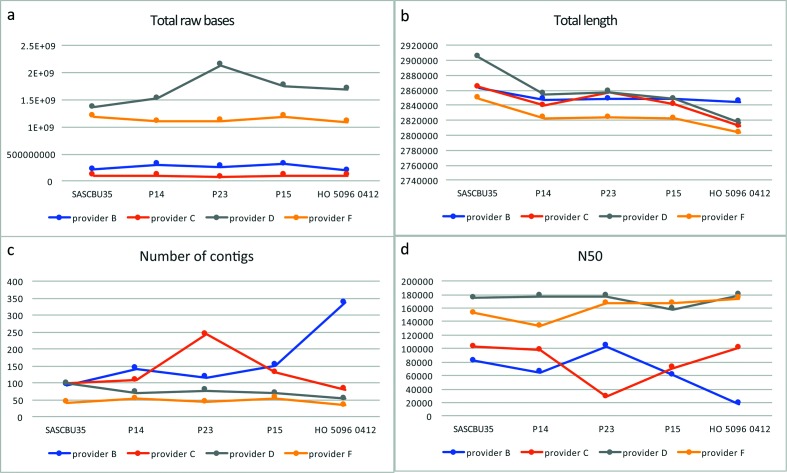
Assembly statistics for sequences returned by the four sequence providers. Graphs showing genome sequence data quality for five MRSA isolates sequenced by four sequence providers. (a) Total number of raw bases returned per isolate, (b) total length of the assembly per isolate, (c) total number of contigs in the assembly per isolate and (d) N50 for the assembly of each isolate (the size of the contig halfway along the assembly).

Analysis of raw data and the results of assembly identified that the four providers had used different read-lengths, ranging from 150 bp (provider F) to 300 bp (provider B) (Table S1). Sequence providers D and F produced more total raw bases and, probably as a result of this, the assemblies from these two providers had a lower number of contigs and a higher N50, signifying a better assembly (Fig. 1). The total number of contigs for the four isolates ranged from 34 to 337 (median 87.5), with two outliers containing a much larger number of contigs than the remainder (P23-provider C, 244 contigs; HO 5096 0412-provider B, 337 contigs) (Fig. 1). The lengths of the assemblies were similar across providers, although provider F produced consistently smaller assemblies and the assembly of isolate SASCBU35-provider D was larger than the remainder (2 903 570 bp versus the next largest at 2 857 410 bp). The results are summarized in Table S1.

### Sequence data analysis

**Fig. 2.**
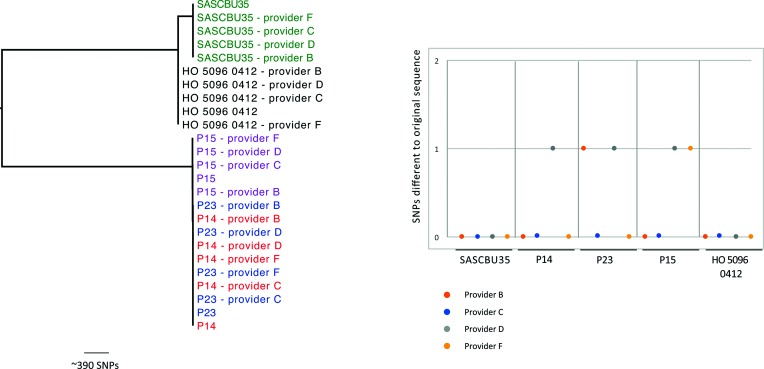
SNPs in the sequences from providers B, C, D and F compared to the original sequence based on mapping to a reference. Left: maximum-likelihood phylogeny of the sequences from each of the four external companies and the sequences from the original study mapped to a reference strain. Labels indicate the isolate names, and colours highlight the five different isolates sequenced. Right: graph indicating the number of SNPs between each sequence returned from the four companies and the corresponding sequence from the original study based on mapping to a reference genome. Colours indicate the provider (orange=C, blue=B, grey=D, yellow=F), and bars along the bottom indicate the four outbreak isolates sent for sequencing.

STs were assigned from the sequence data. One allele could not be assigned for P23 from provider C data (*arcC*) or for reference strain HO 5096 0412 from provider B data (*aroE*). The blast analysis (https://blast.ncbi.nlm.nih.gov/Blast.cgi) demonstrated that this was due to fragmentation of the locus across contigs in both cases, which is consistent with the higher contig number for these two specific isolates from these providers. MRSA outbreak analysis is based on mapping of the genome to a reference, and enumeration of SNPs between test isolates. This was recapitulated for the newly generated data for the four SCBU isolates (P14, P15, P23 and SASCBU35) and reference isolate (HO 5096 0412), in which these were mapped to the original HO 5096 0412 sequence data and then compared with the original sequence for each isolate. This demonstrated that five isolates from three providers had a single additional SNP (Fig. 2) [provider B (*n*=1), D (*n*=3) and F (*n*=1)]. Reasons for these differences could be heterogeneity in the selected colony or differences in the quality of the read data, such as the insert sizes. The insert sizes (the length of the sequence between the adapters) for the data from providers B, C and F were smaller than the combined length of the forward and reverse reads (Table S1), meaning that ~100–200 bp of the DNA fragments were sequenced twice. This could improve the sequence quality at the ends of the reads, where quality usually drops. However, a single SNP difference would lead to no changes in the interpretation of relatedness in the context of an outbreak.

## Discussion

Pathogen genome sequencing for applications such as outbreak investigation and the agnostic detecting of genes encoding antibiotic resistance or virulence factors could bring major improvements to infection control and clinical practice. The availability of sequencing instruments in routine diagnostic microbiology laboratories is currently low, and there are no providers to our knowledge that offer a clinically accredited pathogen sequencing service to unaffiliated users. We investigated whether send-out services were a viable option, focusing on the Illumina MiSeq instrument based on its relatively short run time of around 24 h. Four providers were selected from a total of eight based on cost and overall service turnaround time. Providers were blinded to the study objectives.

The four providers returned sequence data within their stated turnaround time, the actual time varying between 12 and 63 days. Although provider C out-performed the others by at least 16 days, a 12 day turnaround plus the time taken to isolate the organism and extract DNA represents a timescale that would be unlikely to provide information that would have impact on clinical and infection control practice. This is because the longer the turnaround time, the more likely it is that the outbreak will have spread further, making it harder to control, and the more likely it is that patients will have moved, for example, to care facilities or the community. This movement may result in transmission to other vulnerable patient populations and make it harder to decolonize affected individuals. It remains possible that the providers could offer a more rapid service, because they were not given the option to offer an expedited service. However, it is likely that a reduction in the turnaround time from that stated by the provider would impact on the cost per sample. In an ideal world the turnaround time for clinical samples would be 24–48 h as this would allow clinicians and infection control staff to rapidly identify individuals involved in transmission and implement procedures to prevent or halt an outbreak, as opposed to ‘playing catch-up’. However, it is possible that turnaround times of less than 1 week may still have clinical value, particularly if an outbreak is centred on a ward or person.

The cost per sample identified in this study was ~£150 to £900. We requested that the five study MRSA isolates be multiplexed and sequenced in a single run to minimize any delays caused by batching samples from elsewhere. Costs could be decreased by multiplexing more isolates on a single run because the quotes were based on purchasing a whole run, in addition to library prep reagents for each sample. This means that the lowest cost provider may represent a viable option for diagnostic laboratories in healthcare settings where reimbursement is or could become available for pathogen sequencing. Cost-effectiveness studies taking into account morbidity, mortality and healthcare costs are now required to determine the cost at which sequencing would be viable for clinical use based on different clinical scenarios.

Whilst we selected a MiSeq for this study based on its short turnaround time, the results may have differed if other instruments or run requirements were allowed. Alternative platforms available for WGS from commercial providers were the Illumina HiSeq and NextSeq500, both of which have longer run times than the MiSeq. Both platforms sequence a higher number of samples per run and enquiries into use of the HiSeq revealed that samples would need to wait for a run to be filled, increasing the turnaround time. Relaxing the run requirements to allow other samples to be sequenced in the same run would probably have reduced costs but would have increased the turnaround time.

All four providers returned data that were of sufficiently high quality to perform further analyses. The two more expensive providers generated more sequence data, which resulted in the correct ST being assigned due to better assemblies. However, analysis tools such as ARIBA can now detect MLSTs from raw reads [13], meaning that STs could be assigned despite fragmented assemblies. Whilst most sequences were identical between providers, five isolates had a single SNP compared to the reference. In a clinical outbreak scenario a single SNP would not change the interpretation of whether two isolates were part of the same outbreak, indicating data of sufficiently high quality for clinical use.

In order for clinical laboratories to use a commercial provider, the WGS service would ideally need to become accredited by the United Kingdom Accreditation Service. This may be a viable option because some providers have achieved accreditation for other services. However, in the absence of this, validation by local clinical laboratories may be a feasible alternative. Requirements for validation for Public Health England laboratories are described in the ‘UK Standards for Microbiology Investigations. Evaluations, validations and verifications of diagnostic tests’ [14] and include repeatability and reproducibility experiments, analysis of the sensitivity and specificity, and use of control strains.

An alternative option for clinical laboratories would be the introduction of routine WGS into reference laboratories, reducing the space and technical expertise required to run the service. Reference laboratories currently offer tests such as molecular typing, toxin gene detection and resistance testing for *S. aureus*, and have recently begun performing WGS for specific clinical samples: gastrointestinal diseases, suspected outbreaks of importance and *Mycobacterium tuberculosis*. However, results can take weeks to be returned and, as with the providers tested in this study, this turnaround time would need to be improved to allow outbreaks to be investigated prospectively and results to be returned in a clinically useful timeframe.

### Conclusion

In summary, we have shown that external providers are a possible option for diagnostic microbiology laboratories that seek to undertake pathogen sequencing, but improvements will be required in turnaround time. This service will also require accreditation. An unresolved barrier is the availability of software that performs automated interpretation and provides information to users that can be understood by those without informatics training. Further investigations of the cost-effectiveness of pathogen sequencing are urgently required to support decisions to reimburse pathogen sequencing costs.

## Data bibliography

1. Harris SR, Cartwright EJP, Török ME, Holden MTG, Brown NM, *et al*. European Nucleotide Archive; ERR72246, ERR108054, ERR72247, ERR131801 (2013).
2. Holden MT, Hsu LY, Kurt K, Weinert LA, Mather AE, *et al*. European Nucleotide Archive, HE681097 (2013).

## Supplementary Data

43 Supplementary File 1
